# Intentional Weight Loss and Associated Cancer Incidence Among People With Overweight or Obesity: A Systematic Literature Review

**DOI:** 10.1002/edm2.70104

**Published:** 2025-09-13

**Authors:** Chi‐Yin Liao, David Schapiro, Donna Mojdami, Kristin M. Sheffield, Meredith M. Hoog, Raghuvir Keni, Wambui Grace Gathirua‐Mwangi, Hong Kan

**Affiliations:** ^1^ Eli Lilly and Company Indianapolis Indiana USA; ^2^ Eli Lilly Services India Private Limited Bengaluru Karnataka India

**Keywords:** cancer incidence, intentional weight loss, obesity‐associated cancers, systematic review, weight management

## Abstract

**Aims:**

While obesity is linked to increased cancer risk, evidence on the impact of intentional weight loss on obesity‐associated cancers (OACs) is limited. A systematic literature review (SLR) was conducted to assess the association between intentional weight loss and cancer incidence, including overall cancers and 13 OACs, from recent observational studies and clinical trials.

**Methods:**

Studies published between January 2019 and May 2023 were searched within MEDLINE, EMBASE, and CENTRAL. Studies assessing the relationship between intentional weight loss, defined as weight reduction via metabolic‐bariatric surgery (MBS) or lifestyle interventions, and cancer incidence were included. A dual independent review process was used to screen 1954 abstracts and 84 full‐text articles, and to extract data from 18 full studies. All discrepancies were resolved by another reviewer.

**Results:**

Of the 18 studies included, 17 studies were observational, focusing on MBS as the method for achieving weight reduction. One randomised controlled trial examined the effect of intensive lifestyle intervention on weight reduction and found no significant association between intentional weight loss and cancer risk. Intentional weight loss was associated with decreased cancer incidence in 71.4% (*n* = 5/7) of studies for all cancers and 66.7% (*n* = 4/6) of studies for OACs, with reported risk reductions of 11% to 33% and 11% to 41%, respectively. For specific OACs, a greater number of studies indicated that weight reduction was associated with reduced occurrence of endometrial (4/4, 100%, 31%–53% risk reduction), female breast (5/9, 55.6%, 19%–50% risk reduction) and colorectal (4/7, 57.1%, 20%–60% risk reduction) cancers.

**Conclusions:**

This SLR highlights the potential cancer risk‐reduction benefit of weight reduction for people with obesity.

## Graphical abstract

We conducted an SLR of recent observational studies and clinical trials to investigate the association between intentional weight loss and cancer incidence, including overall cancers and 13 OACs. Of the 18 studies included, intentional weight loss was associated with decreased cancer incidence in 71.4% (*n* = 5/7) of studies for overall cancers and 66.7% (*n* = 4/6) of studies for OACs, with reported risk reductions of 11% to 33% and 11% to 41%, respectively. For specific OACs, a greater number of studies indicated that weight reduction was associated with reduced occurrence of endometrial (4/4, 100%, 31%–53% risk reduction), female breast (5/9, 55.6%, 19%–50% risk reduction) and colorectal (4/7, 57.1%, 20%–60% risk reduction) cancers. This SLR highlights the potential cancer risk‐reduction benefit of intentional weight loss for people with obesity.

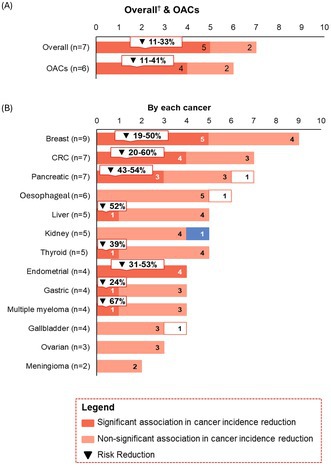

## 1 Introduction

It was estimated that around 2.5 billion adults worldwide were living with overweight or obesity in 2022 [1]. Obesity is known to contribute to approximately 4%–8% of all cancers [2, 3] and is specifically recognised as a risk factor for 13 distinct types of cancers [4, 5]. It increases the risk of cancer through chronic inflammation, insulin resistance, altered adipokine levels, and hormonal imbalances [6]. In the US, most cancers associated with obesity have risen, especially among women, while cancers related to other causes have declined [5, 7]. With obesity ranking as the second most common preventable cause of cancer, a Clinical Practice Statement established by the Obesity Medicine Association in 2022 recommends weight management as a potential option to reduce the risk of cancer onset, progression, or recurrence in people with obesity [8, 9].

A large body of literature has indicated a correlation between high BMI or excess body fat and an increased rate of cancer incidence [10, 11, 12, 13, 14, 15, 16, 17, 18, 19, 20, 21, 22, 23, 24, 25, 26]. However, studies evaluating the relationship between weight loss and reduced cancer risk have yielded inconsistent results [27, 28, 29], possibly due to the varying causes behind the weight loss in these studies. Unintentional weight loss is associated with underlying comorbidities that could be a symptom of cancers [30, 31]. In contrast, intentional weight loss, typically achieved through lifestyle modifications (such as a diet with reduced calories combined with exercise), behavioural changes, obesity medications, and/or weight reduction surgery, may favourably impact metabolic and hormonal functions, thereby potentially reducing the risk of developing cancers.

To understand the effect of intentional weight loss on cancer incidence, studies have primarily focused on the effect of metabolic‐bariatric surgery (MBS), a surgical procedure that is performed in people with obesity to provide sustained weight reduction [32, 33], on overall and obesity‐related cancer incidences. However, results are mixed with some studies suggesting MBS decreased cancer risk [34, 35] and some suggesting no difference [36, 37]. Systematic literature reviews (SLRs) or meta‐analyses (MAs) that synthesise the evidence on the relationship of intentional weight loss and cancer incidence are limited [38, 39, 40].

This SLR, as a recent literature update, aims to assess the association between intentional weight loss through MBS and cancer incidence, based on the most recent literature among individuals with obesity over the past five years. Given that findings from MBS studies may have limited applicability due to the stringent eligibility criteria for people with very high BMI and/or those with complications, this SLR seeks to incorporate studies that employ lifestyle weight loss interventions to bridge the current knowledge gap. Specifically, this study will identify research evaluating intentional weight loss through all interventions among people living with overweight or obesity. The associated absolute and relative incidence of all cancers, overall obesity‐associated cancers (OACs), and each of the 13 specific OACs – including both haematological malignancy (multiple myeloma) and solid tumour malignancies (endometrial, oesophageal, gastric, liver, kidney, pancreatic, colorectal, gallbladder, post‐menopausal breast, ovarian and thyroid cancers as well as meningioma) [4, 5] – will be reported.

## 2 Methods

This SLR review followed the recommendations of the Center for Reviews and Dissemination, Preferred Reporting Items for Systematic Reviews and Meta‐Analyses (PRISMA) statement (Table S3). The OVID platform was used to search for peer‐reviewed studies across data sources from the Medical Literature Analysis and Retrieval System Online (MEDLINE), Excerpta Medica Database (EMBASE) and Cochrane Central Register of Controlled Trials (CENTRAL). To incorporate the most recent evidence, grey literature from relevant conferences was also searched from EMBASE through the Ovid platform, or manually searched from the conference websites when records were not available from EMBASE. Outcomes included overall cancer incidence and specific cancer incidence for 13 obesity‐related cancers.

### 2.1 Publication Search

The search was limited to studies published since January 1, 2019 to May 4, 2023. As a literature update from a recent MA [40], this study further expands weight loss interventions to lifestyle interventions, including diet and physical activity. In addition to peer‐reviewed studies, we also searched grey literature from relevant conferences, including ObesityWeek, Annual Meeting of the Endocrine Society, Scientific Sessions of the American Diabetes Association and European Congress on Obesity, to incorporate the most recent evidence in the past 2 years (Table S1). EMBASE was used when abstracting data from the relevant conferences. If the published supplement that contained the conference abstracts was not indexed, a manual search from the conference website was conducted. Posters or presentation slides were further obtained from conference websites.

### 2.2 Literature Screening and Eligibility Criteria

The literature screening and study selection for this paper were based on specific eligibility criteria. The studies must be full‐text articles written in English and published from January 2019 to May 2023. Eligible studies were included on PICOS criteria. The population of interest (P) includes adults (age ≥ 18) with overweight or obesity. The intervention (I) involves the patient population participating in intentional weight loss interventions resulting in weight change. An intervention is defined as intentional if weight loss is achieved through surgical (MBS) or non‐surgical lifestyle change interventions (diet or exercise). Self‐reported data on intentionality are also acceptable. Weight loss/change is defined as a change in weight/BMI/other measures of adiposity. Information on weight loss/change is required for the study to be eligible, unless the weight loss/change is achieved through MBS, which is assumed to lead to sustained weight reduction for most people.

The outcomes (O) of interest are cancer incidence outcomes. These include the absolute incidence rate by groups with different levels of weight/BMI change and the relative difference reported as hazard ratio, risk ratio, or odds ratio between groups with different levels of weight/BMI change. The outcomes can be overall cancer incidence, cancer incidence of a combination of obesity‐related cancers, or cancer incidence for any of the 13 specified obesity‐related cancers. These include endometrial, oesophageal, gastric, liver, kidney, pancreatic, colorectal, gallbladder, post‐menopausal breast, ovarian, and thyroid cancers, as well as multiple myeloma and meningioma. The absolute risk refers to the absolute cancer incidence rate within the group with weight reduction and the group without, while the relative risk refers to the difference or ratio between the weight reduction versus weight maintained, or weight reduction versus weight increase. The study designs (S) of interest are randomised controlled trials, non‐randomised intervention studies, or observational studies.

The following exclusion criteria (E) were used for selecting literature in this SLR: Studies focusing solely on patients with a prior cancer diagnosis (studies on cancer survivors, or studies that did not exclude the prior cancer diagnosis that is being investigated by the study), and those focusing on paediatric, adolescent, or pregnant patients were excluded. Studies were also excluded if the weight loss was unintentional or if the intentionality could not be determined, especially in observational studies. For lifestyle change intervention studies, exclusions applied to those not reporting changes in weight/BMI or other obesity‐relevant measures. Other exclusions include case reports or case series, single group designs, cross‐sectional designs, comments and opinions, letters and editorials, books or book chapters, guidelines, consensus statements, SLRs, MAs, narrative reviews, pilot studies, and articles investigating in vitro, animal, foetal, molecular, genetic, pathologic, or pharmacokinetic, pharmacodynamic outcomes.

### 2.3 Search Strategy

The search strategy for this study involved a comprehensive search of the PubMed, Cochrane, and Embase databases. The search focused on articles published in recent years that explored the association between weight reduction and cancer incidence among people with obesity. The strategy was developed according to the objective of this SLR.

The search strategies combined both free text and controlled vocabulary terms, such as Medical Subject Headings in MEDLINE and CENTRAL, and EMTREE terms in EMBASE. These terms were related to the disease and treatment, including “Bariatric Surgery,” “Obesity,” and “Malignant Neoplasm or Cancers”. To enhance the sensitivity of the search, synonyms for “Body Weight Changes” and “Weight Loss” were utilised. Eligible study designs and exclusion terms were applied to further refine the study records and identify relevant studies. Detailed search results are provided in Table S2.

### 2.4 Study Selection

The study selection process adhered to the PRISMA guidelines, and a PRISMA flow chart was reported (Table S3) [41]. Records identified from the databases were combined, and duplicates were removed. The screening of eligible studies was conducted at two levels by two independent reviewers (C.‐Y.L. and R.K.). At the first level, the title and abstract of all identified records were screened according to the eligibility criteria. At the second level, the full text of the peer‐reviewed studies was retrieved for a more detailed eligibility assessment. Any discrepancies between the two reviewers were resolved, and a consensus was reached with the involvement of a third team member.

### 2.5 Data Extraction

Data from included studies that met the eligibility criteria were extracted by two independent reviewers. Discrepancies between the two reviewers were resolved by involving a third team member. Data extractors were not blinded to any study information. Before data extraction began, a standardised data extraction form was developed based on discussion with study team members on variables to be extracted. Key study variables were recorded onto the data extraction grid, including study characteristics, treatment/intervention characteristics, and outcomes.

### 2.6 Bias Assessment

Biases were assessed by one reviewer and verified by a second reviewer, with discrepancies being resolved by a third team member. Data on study quality were extracted simultaneously with other study‐related data. When a quality assessment question did not apply, it was reported as not applicable (NA). At the time when data regarding study quality was extracted, reviewers were prompted to make comments regarding the potential implications for each identified source of potential bias on the study results. Cochrane Risk of Bias Tool v2.0 was used for RCTs, while Newcastle Ottawa Scale (NOS) was used for non‐randomised studies, including non‐randomised trials and observational studies.

## 3 Results

**FIGURE 1:**
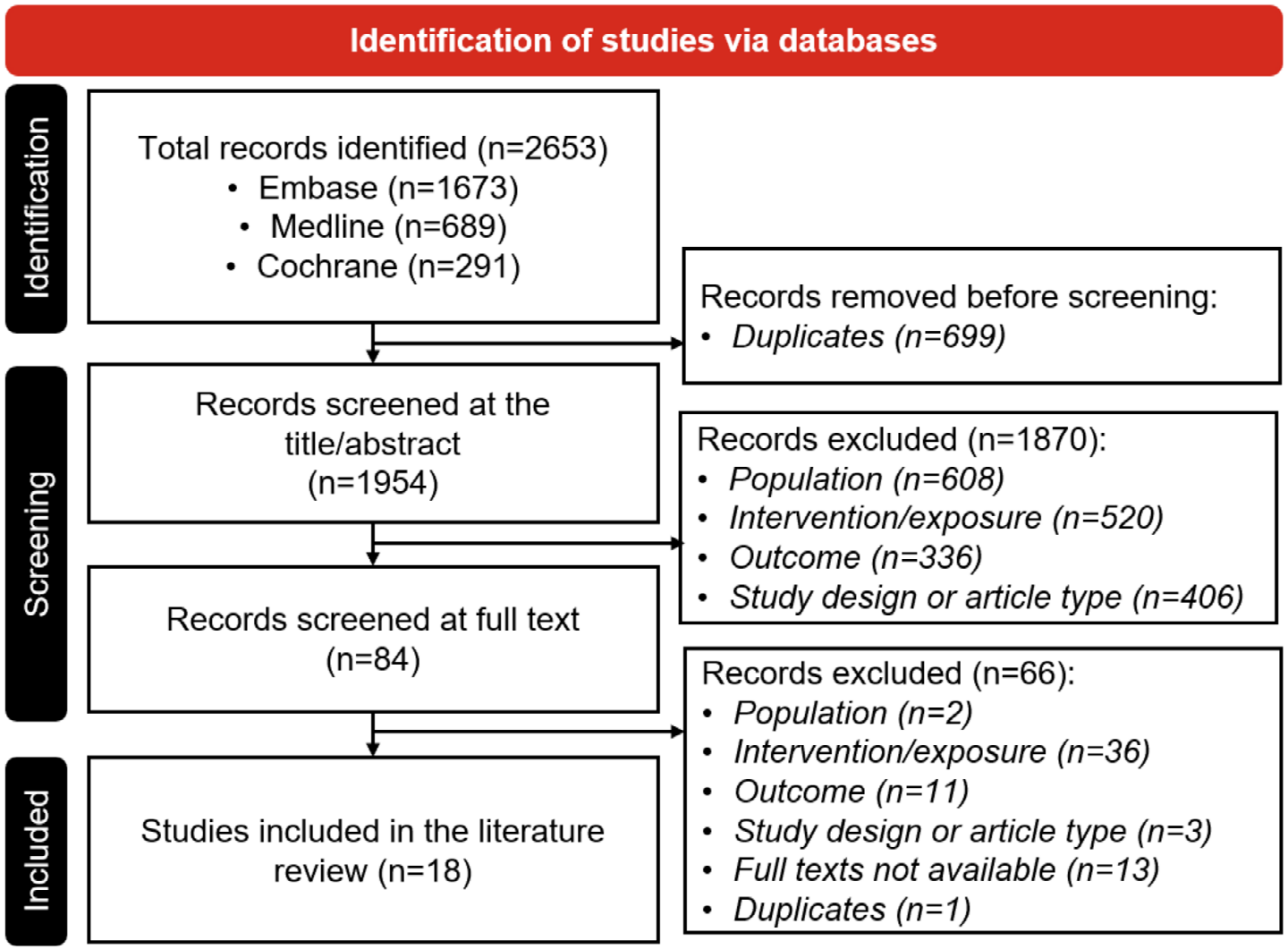
PRISMA Flowchart.

Figure 1 illustrates the study identification process. Initially, a total of 2653 records were identified through database searches, with Embase contributing 1673 records, Medline 689, and Cochrane 291. Prior to screening, 699 duplicate records were removed. At level one, 1954 records were screened for the title and abstract. At level two, 84 records underwent full‐text assessment. Initially, 61 records were excluded. A third reviewer was involved to exclude an additional 5 records: one reporting surrogate cancer outcomes instead of actual cancer diagnosis [42], two with indeterminate weight loss intentionality [27, 43], one using the age of surgery rather than weight reduction as exposure [44], and one study with an irrelevant study design [45]. This resulted in a total of 66 exclusions for various reasons: 2 due to population, 36 due to intervention/exposure, 11 due to outcome, and 3 due to study design or article type. Additionally, 13 full texts were not available, and 1 duplicate was found. Ultimately, 18 studies were included in the qualitative synthesis [46, 47, 48, 49, 50, 51, 52, 53, 54, 55, 56, 57, 58, 59, 60, 61, 62, 63].

Table 1 summarises the characteristics of studies included in this SLR on the impact of weight loss interventions on cancer incidence. Of these studies, 17 out of 18 were observational studies that focused on MBS as a weight loss intervention, while only 1 study evaluated the effect of intensive lifestyle intervention incorporating exercise and diet. The weight loss interventions examined in these studies included different types of bariatric surgeries (BS) such as Roux‐en‐Y gastric bypass (RYGB), sleeve gastrectomy (SG), adjustable gastric banding (AGB), and others, compared against non‐surgical interventions (NS) or general population (GP) controls. Except for one study that compared the cancer risk between the surgical intervened individuals and the general population that may not have obesity, all remaining studies included a control group or a subgroup consisting of individuals with obesity. Only 7 studies reported a mean or median follow‐up length of more than 5 years, and only 4 studies reported weight change for the study participants. In terms of geography, the studies are predominantly conducted in North America (9 studies, including 3 in Canada and 6 in the US) and Europe (7 studies, including 3 in France, 1 in Italy, and 3 in Nordic countries), with a smaller number in Asia (2 studies).

**TABLE 1:** Study characteristics.

Study	Study design	Follow‐up length	Geography	Weight loss intervention	Weight loss data	Cancer incidence reported^a^
Doumouras et al. [46]	OS	NR	Canada	BS: RYGB and SG vs. NS: 1:1 matched for each BMI group	NR	Breast cancer (not specified pre‐ or post‐menopausal)
Bulsei et al. [47]	OS	5+ years	France	BS: AGB, RYGB, and SG vs. NS: with obesity	NR	Pancreatic cancer
Lazzati et al. [48]	OS	5+ years	France	BS: AGB, SG, gastric bypass, and biliopancreatic diversion vs. NS: with obesity	NR	Oesophageal and gastric cancers
Hussan et al. [49]	OS	< 5 years	US	BS: RYGB or SG vs. NS: with obesity	NR	CRC
Aminian et al. [50]	OS	5+ years	US	BS: RYGB or SG vs. NS: with obesity	Reported	Overall, OACs, specific cancers
Doumouras et al. [51]	OS	NR	Canada	BS: RYGB or SG vs. NS: with obesity	NR	Breast cancer (not specified pre‐ or post‐menopausal)
Ciccioriccio et al. [52]	OS	< 5 years	Italy	BS: RYGB or SG vs. GP: may not have obesity	NR	CRC
Wei et al. [53]	OS	< 5 years	Hong Kong	BS: LSG, LGBP, LAGB vs. NS: with obesity	Reported	Overall, OACs, specific cancers
Kao et al. [54]	OS	5+ years	Taiwan	BS (types NR) vs. GP: without obesity NS: with obesity vs. GP: without obesity	NR	Overall, specific cancers
Rustgi et al. [55]	OS	< 5 years	US	BS: RYGB, laparoscopic RYGB, SG, biliopancreatic diversion‐DS, LSG, LAGB, or other partial gastrectomy vs. NS: with obesity	NR	Overall, OACs, specific cancers
Andalib et al. [56]	OS	5+ years	Canada	BS1: RYGB (Reflux‐protective) BS2: SG&DS (Reflux‐prone) vs. NS: with obesity	NR	Oesophageal cancer
Taube et al. [57]	OS	< 5 years	Sweden	BS: GB, banding or vertical banded gastroplasty vs. NS: with obesity	Reported	CRC, colon, and rectal cancer
Tao et al. [58]	OS	< 5 years	Denmark, Finland, Iceland, Norway, and Sweden	BS: GB, Restrictive and other surgery vs. NS: with obesity	NR	Overall, OACs, specific cancers
Yeh et al. [59]	RCT	5+ years	US	Intensive lifestyle intervention vs. Diabetes support and education	Reported	Overall, OACs, specific cancers
Tao et al. [60]	OS	< 5 years	Denmark, Finland, Iceland, Norway, and Sweden	BS: GB, Restrictive and other surgery vs. NS: with obesity	NR	Colon and rectal cancers
Bailly et al. [61]	OS	5+ years	France	BS: AGB, GB, and SG vs. NS: with obesity	NR	CRC
Feigelson et al. [62]	OS	< 5 years	US	BS: RYGB, LAGB, and SG vs. NS: with obesity	NR	Pre‐ and post‐menopausal breast cancer
Schauer et al. [63]	OS	< 5 years	US	BS: GB, SG, LAGB vs. NS: with severe obesity	NR	Overall, OACs, specific cancers

Abbreviations: AGB, adjustable gastric banding; BS, bariatric surgery intervention; CRC, colorectal cancer; DS, duodenal switch; GP, general population; GB, gastric bypass; LAGB, laparoscopic adjustable gastric banding; LGB, laparoscopic gastric bypass; LSG, laparoscopic sleeve gastrectomy; NR, not reported; NS, non‐surgical intervention; OACs, obesity‐associated cancer; OS, observational study; RYGB, Roux‐en‐Y gastric bypass; SG, sleeve gastrectomy.

^a^ The term “overall” refers to instances when the study did not focus on specific type/s of cancer and the definition (excluding skin cancers/melanoma) varied from study to study.

### 3.1 Cancer Outcome

Of the 18 studies included, 7 studies reported incidence for overall cancers; of these 7 studies, 6 studies also reported incidence for OACs. A total of 11 studies reported incidence only for specific OACs. For specific cancers, the most commonly reported cancer incidences are breast cancer (*n* = 9), which is followed by colorectal cancer (*n* = 7) and pancreatic cancer (*n* = 7).

**FIGURE 2:**
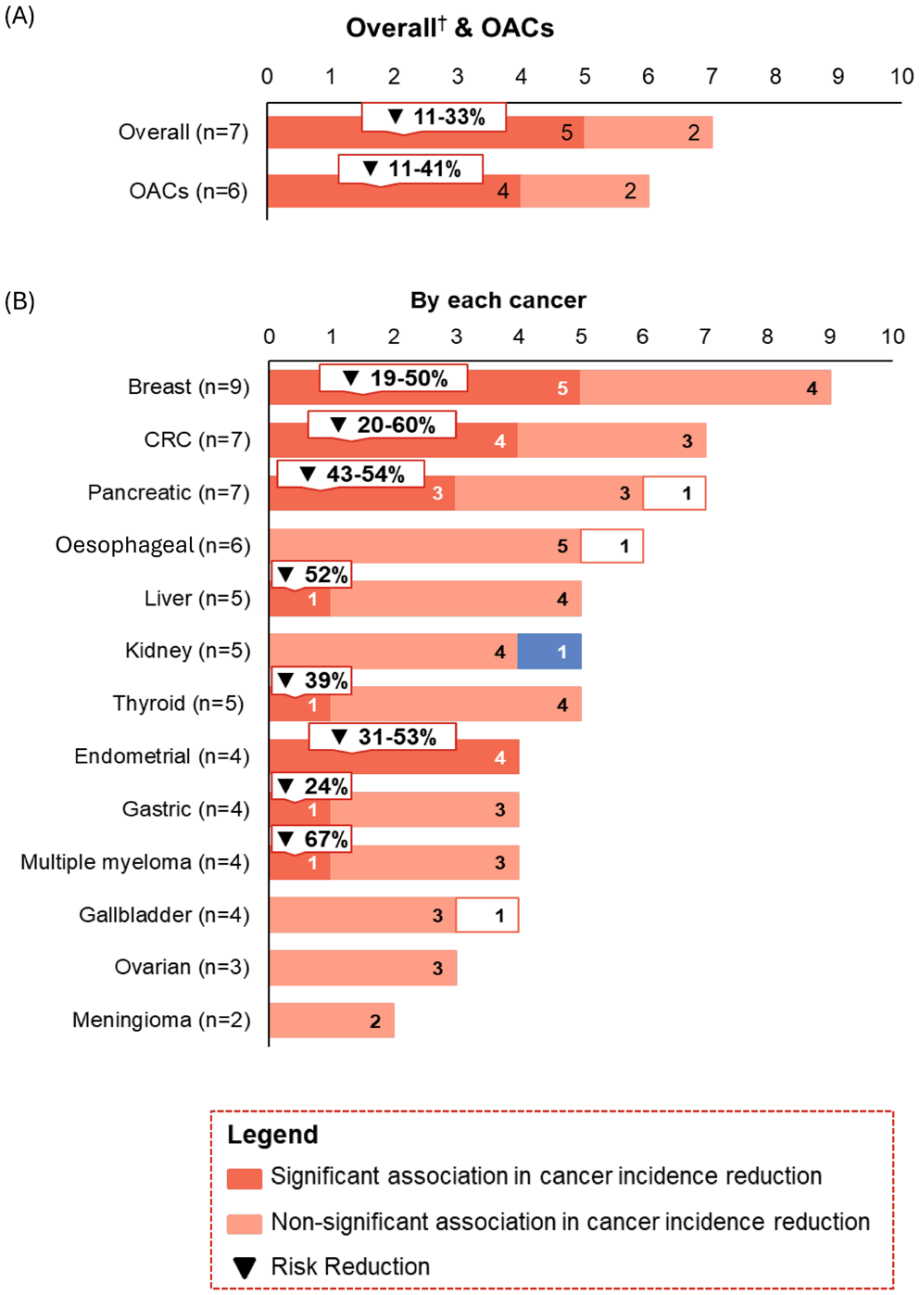
Cancer incidences results: (A) overall and OACs; (B) by each cancer. CRC, colorectal cancers; OACs, obesity‐associated cancers. The presented % is based on point estimates of relative risk measure reported across studies with statistical significance findings; risk reduction % was calculated using the following hierarchy: adjusted hazard ratio (aHR), standardised incidence ratio (SIR) and absolute risk difference. For example, if aHR = 1.20 for the control group versus the weight loss group, or if SIR = 0.8 for the weight loss group versus the control group, risk reduction is noted as 20%. ^†^The term overall refers to instances when the study did not focus on specific type/s of cancer and the definition (excluding skin cancers/melanoma) varied from study to study.

For overall cancers, a total of 5/7 (71.4%) showed that intentional weight loss was significantly associated with a reduced incidence, with reported risk reductions ranging from 11% to 33% (Figure 2A). On the other hand, 2/7 (28.6%) did not find a difference [53, 59]. For OACs, the association between intentional weight loss and reduced cancer incidence was found to be significant in 4/6 (66.7%) studies, with risk reduction ranging from 11% to 41% (Figure 2A). The other two studies (33.3%) found no significant difference for the overall population [53, 59]. Two studies conducted stratification analysis by gender, and found significant risk reductions were only observed among women [58, 63].

Of specific OACs, endometrial (4/4, 100%, 31%–53% risk reduction), female breast (5/9, 55.6%, 19%–50% risk reduction), and colorectal cancers (4/7, 57.1%, 20%–60% risk reduction) were the three OACs with a greater number of studies demonstrating the significant association between intentional weight loss and decreased cancer risk (Figure 2B). All studies reporting incidence for endometrial cancer showed consistent, significant cancer risk‐reduction results (Table S4D) [50, 55, 58, 63]. For breast cancer (*n* = 9, with 4 did not specify pre‐ or post‐ menopausal cases [50, 55, 59, 63], 4 focused only on post‐menopausal cases [46, 51, 54, 58], and 1 included both pre‐ or post‐ menopausal [62]), of the 5 demonstrating the significant breast cancer risk reduction, 3 studies did not specify pre‐ or post‐ menopausal cases [46, 51, 58], 1 study was based on a post‐menopausal population [63]; the remaining 1 study included both pre‐ and post‐menopausal status [62], and the significant association was found within both pre‐ and post‐menopausal stratifications (Table S4C). For colorectal cancers (CRC) (*n* = 7), of 4 studies that reported a significantly lower incidence of colorectal cancer in people with intentional weight loss [49, 52, 55, 61], one study reported a significant inverse association among women with RYGB versus women without surgery [49]; the other study found a significant inverse association across gender and surgical procedure strata, except for men with RYGB (Table S4I) [52]. For the remaining specific OACs, the decreased incidence associated with intentional weight loss was found in 3/7 studies reporting for pancreatic cancer [47, 55, 63], 1/5 study for liver [55], 1/5 for thyroid [55], 1/4 for gastric [48] and 1/4 for gastric48 and 1/4 for multiple myeloma [55] (Figure 2B).

### 3.2 Results of Bias Assessment

The majority of the observational studies received high‐quality ratings (6–9), with only four studies being rated as fair (Table 2). As for the RCT, the randomisation process, outcome measurement, and reported results selection were all deemed to have a “low” risk of bias [59]. The risk was considered “relatively low” for missing outcomes data, while there was “some concern” regarding deviations from intended interventions.

**TABLE 2:** Results of bias assessment.

	Selection (total 4)	Outcome (total 3)	Comparability (Total 2)	Total score	Rating^a^
Doumouras et al. [46]	4	1	1	7	High
Bulsei et al. [47]	4	2	3	8	High
Lazzati et al. [48]	4	2	2	7	High
Hussan et al. [49]	4	1	1	6	High
Aminian et al. [50]	3	2	2	7	High
Doumouras et al. [51]	4	1	1	7	High
Ciccioriccio et al. [52]	2	1	1	3	Fair
Wei et al. [53]	3	1	2	7	High
Kao et al. [54]	3	2	2	5	Fair
Rustgi et al. [55]	4	1	1	6	High
Andalib et al. [56]	3	2	2	7	High
Taube et al. [57]	4	2	2	7	High
Tao et al. [58]	3	1	1	5	Fair
Yeh et al. [59]^b^	NA	NA	NA	NA	NA
Tao et al. [60]	4	1	1	5	Fair
Bailly et al. [61]	4	2	2	7	High
Feigelson et al. [62]	4	1	1	6	High
Schauer et al. [63]	4	1	1	7	High

*Note:* Risk of bias was assessed as “low” for the randomisation process, measurement of outcome and selection of reported results, as “relatively low” for missing outcomes data and as “some concern” for deviation from intended interventions.

Abbreviation: NA, not available.

^a^ 0–2 (poor quality), 3 to 5 (fair quality), 6 to 9 (good/high quality).

^b^ Cochrane revised tool for Risk of Bias (RoB 2) in randomised trials was used.

## 4 Discussion

Our study updated the most recent literature from January 2019 to May 2023 on intentional weight loss and its impact on cancer incidence in people with obesity. This SLR further reinforces existing knowledge that, in people with obesity, intentional weight loss was associated with decreased incidence of overall cancer and OACs. The evidence is particularly strong for breast, endometrial, and colorectal cancers. Overall, our findings suggest that there are potential cancer risk‐reduction benefits associated with intentional weight loss in people with obesity.

Our study results align closely with recently published findings. Our findings on the decreased incidence of overall cancer and OACs associated with weight reduction are consistent with findings from a recent cohort study with 10 to 11 years of median follow‐up [64]. The decreased incidence for breast and endometrial cancers summarised in our study also mirrors the findings of a previous meta‐analysis that showed a 51% risk reduction in breast cancer (OR = 0.49, 95% CI = 0.33–0.72) and a 57% reduction in endometrial cancer (OR = 0.43, 95% CI = 0.26–0.71) among people who underwent MBS [65].

Notably, among the 18 studies examined, only one RCT targeted a lifestyle‐based weight loss intervention [59]. Despite significant weight loss from the intensive lifestyle intervention, no substantial difference was observed in overall, OAC, and specific cancers between the intervention and control groups [59]. The lack of positive outcomes may be partly attributed to the transient or modest effects of lifestyle‐based weight loss interventions [66], which also contribute to the scarcity of such studies in assessing their impact on cancer incidence.

The findings from this SLR suggest a promising avenue for cancer prevention strategies, with more evidence expected from several ongoing trials investigating the link between MBS and the prevention of recurrent breast and endometrial cancers [67, 68, 69, 70]. While this current SLR focused on the impact of surgery and lifestyle modifications on cancer risk reduction, with the recent approval of obesity management medications, including dual glucose‐dependent insulinotropic polypeptide and glucagon‐like peptide‐1 (GIP/GLP‐1) receptor agonist (RA), there is a growing interest to explore their potential benefits on cancer prevention associated with weight reduction. Despite the concerns about the potential carcinogenic effect of GLP‐1 RA found in preliminary and in vitro studies [71, 72, 73], recent MAs have demonstrated that tirzepatide and semaglutide were not associated with an increased risk of any type of cancer [74, 75]. Further, the cancer prevention effect has been supported by a recent retrospective cohort study that showed the protective effects of GLP‐1 receptor agonists against CRC among people with type 2 diabetes and obesity [76].

Emerging evidence suggests that obesity contributes to cancer development through several interconnected mechanisms that vary by cancer type [6]. For instance, chronic low‐grade inflammation is a key driver that disrupts immune signalling (i.e., promoting polarisation of M1 macrophages [77] and release of proinflammatory cytokines [78]) and induces hypoxia in the adipose tissue. The proinflammatory microenvironment is particularly linked to the development of pancreatic, oesophageal, colorectal, renal, and liver cancers [79]. On the other hand, hormonal imbalances, including elevated oestrogen levels and insulin resistance, are more strongly associated with hormone‐sensitive cancers. Oestrogen excess increases the risk of breast and endometrial cancers, while insulin resistance activates the insulin/IGF‐1 pathway, promoting colorectal cancer progression [79]. These underlying mechanisms provide biological plausibility that weight reduction strategies, by treating obesity, may reduce cancer risk through attenuating obesity‐induced pro‐cancer pathways relevant to each cancer type.

Heterogeneity exists across the studies reviewed. The length of the washout period (the period of time from intervention to the time when the occurrence of cancer is counted as an event) and the follow‐up period differed across studies, which could result in inconsistent results between studies. Detailed information on the primary site, stage, or grade was also frequently unavailable and inconsistently reported. Further, the exclusion of prior malignancy was also handled differently across studies: some excluded patients with a history of the specific cancer that was the study's primary focus, while others – particularly those examining multiple cancer types – excluded individuals with any prior history of malignancy. These inconsistencies may introduce bias, as a history of any primary malignancy can increase the risk of subsequent cancers. Additionally, among studies evaluating MBS, the types of procedures, while most commonly RYGB and SG, have varied across studies. Although both procedures are effective for weight reduction, SG is associated with a lower risk of complications but may increase the risk of gastroesophageal reflux [80, 81], which is a risk factor for oesophageal and gastric cancer. This may coexist with the protective effects of weight loss on cancer risk following SG, limiting the ability to draw definitive conclusions on the oesophageal and gastric cancer risk.

In addition to heterogenicities across studies reviewed, our SLR has several limitations. Firstly, the findings may have limited generalizability, especially for individuals with overweight or who have undergone lifestyle‐based weight loss interventions. This is partly because the majority of the studies included were based on MBS, which is typically performed on people with severe obesity. Additionally, the cancer prevention pathways may not be exactly the same between MBS and lifestyle‐related weight loss, as the physiological changes from the surgical procedure may independently affect cancer risk [82]. Most of the studies reviewed were conducted in developed countries and may have limited applicability to populations in developing countries. Additionally, studies included in this review may have been subject to measured and unmeasured confounding. Studies rarely adjusted for socioeconomic factors and health behaviours following the weight loss intervention, such as smoking, diet, and physical activity. People who undergo MBS often have more severe disease and a higher screening rate, potentially leading to more diagnosed cases. Thus, the effect of intentional weight loss may be underestimated.

Upon review, several directions for future research are identified. As only four studies reported post‐intervention weight loss data, this leaves the magnitude of weight reduction associated with reduced cancer risk unclear. The relative incidence of specific cancers of OACs, such as ovarian cancer and meningioma, was reported in only three studies [50, 55, 63] and two studies [50, 55], respectively. More studies are warranted to shed light on the dose–response relationship between intentional weight loss and cancer risk reduction, especially for OACs with rare occurrence. Additionally, this SLR only included one study evaluating non‐surgical weight reduction [59] and we did not include studies evaluating obesity management medications. Future research should evaluate the impact of intentional weight loss from intensive lifestyle interventions and obesity management medications. This is especially true for next‐generation obesity management medications that are providing weight reduction efficacy nearing that of MBS. Studies that evaluate the association between broader exposure to obesity management medications and cancer incidence with extended follow‐up time will be warranted.

## 5 Conclusion

Intentional weight loss was significantly associated with cancer risk reductions in overall cancers, OACs, and specific cancers, including endometrial, female breast, and colorectal cancers, in the majority of studies identified. Given the recent increase in the incidence of OACs, particularly among women, weight loss intervention warrants consideration as a strategy for cancer risk reduction. Clinicians should consider the importance of weight management for cancer risk reduction among people with obesity. Public health initiatives that raise awareness about the link between obesity and cancer risk and promote weight management and obesity treatment as a preventive measure may be valuable as part of broader health campaigns.

## Author Contributions

**Chi‐Yin Liao:** formal analysis (lead), investigation (lead), methodology (lead), project administration (lead), visualization (lead), writing – original draft (lead), writing – review and editing (lead). **David Schapiro:** methodology (supporting), writing – review and editing (supporting). **Donna Mojdami:** writing – review and editing (supporting). **Kristin M. Sheffield:** writing – review and editing (supporting). **Meredith M. Hoog:** methodology (supporting), writing – review and editing (supporting). **Raghuvir Keni:** formal analysis (supporting), methodology (supporting), writing – original draft (supporting), writing – review and editing (supporting). **Wambui Grace Gathirua‐Mwangi:** writing – review and editing (supporting). **Hong Kan:** conceptualization (lead), supervision (lead), writing – review and editing (supporting).

## Ethics Statement

The authors have nothing to report.

## Conflicts of Interest

At the time of the study, all authors reported employment at Eli Lilly and Company and its subsidiaries.

## Supporting information


**Table S1:** List of conferences searched for grey literature.
**Table S2:** Search strategy.
**Table S3:** Preferred reporting items for systematic reviews and meta‐analyses (PRISMA) statement.
**Table S4A:** Association between intentional weight loss and overall cancer incidence, by each study (*n* = 7).
**Table S4B:**. Association between intentional weight loss and a combination of obesity‐related cancer risk, by each study (*n* = 6).
**Table S4C:** Association between intentional weight loss and female breast cancer risk, by each study (*n* = 9).
**Table S4D:** Association between intentional weight loss and cancer risk for endometrial and ovarian cancer, by each study (*n* = 3 for a combination of female breast and genital organ cancer, *n* = 3 for endometrial cancer, *n* = 3 for ovarian cancer).
**Table S4E:** Association between intentional weight loss and cancer risk for oesophageal cancer and gastric cancer, by each study (*n* = 2 reported a combination of oesophageal, gastric and other digestive organ cancers, *n* = 6 for oesophageal, *n* = 4 for gastric).
**Table S4F:** Association between intentional weight loss and cancer risk for liver cancer and gallbladder cancer, by each study (*n* = 1 for liver or gallbladder, *n* = 5 for liver, *n* = 4 for gallbladder).
**Table S4G:** Association between intentional weight loss and cancer risk for kidney cancer, by each study (*n* = 5).
**Table S4H:** Association between intentional weight loss and cancer risk for pancreatic cancer, by each study (*n* = 7).
**Table S4I:**. Association between intentional weight loss and cancer risk for colorectal cancer, by each study (CRC: *n* = 7, colon: *n* = 5 [4 with relative incidence measure], rectum/anus: *n* = 4 [3 with relative incidence measure]).
**Table S4J:** Association between intentional weight loss and cancer risk for thyroid cancer, by each study (*n* = 5).
**Table S4K:** Association between intentional weight loss and cancer risk for multiple myeloma, by each study (*n* = 4).
**Table S4L:** Association between intentional weight loss and cancer risk for meningioma, by each study (*n* = 2).

## Data Availability

All data analysed during this systematic literature review are published as listed in references, and relevant collated data are included in this article [and/or] its Supporting Information.
