# Supplementary material for: Intentional Weight Loss and Associated Cancer Incidence Among People With Overweight or Obesity: A Systematic Literature Review
Source: Endocrinol Diabetes Metab. 2025 Sep 13;8(5):e70104. doi: 10.1002/edm2.70104 (PMC12432335; doi:10.1002/edm2.70104)
Supplement: Supplementary file 1 — Table S1: List of conferences searched for grey literature. Table S2: Search strategy. Table S3: Preferred reporting items for systematic reviews and meta‐analyses (PRISMA) statement. Table S4A: Association between intentional weight loss and overall cancer incidence, by each study (n = 7). Table S4B:. Association between intentional weight loss and a combination of obesity‐related cancer risk, by each study (n = 6). Table S4C: Association between intentional weight loss and female breast cancer risk, by each study (n = 9). Table S4D: Association between intentional weight loss and cancer risk for endometrial and ovarian cancer, by each study (n = 3 for a combination of female breast and genital organ cancer, n = 3 for endometrial cancer, n = 3 for ovarian cancer). Table S4E: Association between intentional weight loss and cancer risk for oesophageal cancer and gastric cancer, by each study (n = 2 reported a combination of oesophageal, gastric and other digestive organ cancers, n = 6 for oesophageal, n = 4 for gastric). Table S4F: Association between intentional weight loss and cancer risk for liver cancer and gallbladder cancer, by each study (n = 1 for liver or gallbladder, n = 5 for liver, n = 4 for gallbladder). Table S4G: Association between intentional weight loss and cancer risk for kidney cancer, by each study (n = 5). Table S4H: Association between intentional weight loss and cancer risk for pancreatic cancer, by each study (n = 7). Table S4I:. Association between intentional weight loss and cancer risk for colorectal cancer, by each study (CRC: n = 7, colon: n = 5 [4 with relative incidence measure], rectum/anus: n = 4 [3 with relative incidence measure]). Table S4J: Association between intentional weight loss and cancer risk for thyroid cancer, by each study (n = 5). Table S4K: Association between intentional weight loss and cancer risk for multiple myeloma, by each study (n = 4). Table S4L: Association between intentional weight loss and cancer risk for [file EDM2-8-e70104-s001.docx]

**Supplementary Table S1. List of conferences searched for grey literature**

1. 40th Annual Meeting of the Obesity Society at Obesityweek® November 1-4, 2022: oral and poster abstracts were published as supplement of Obesity (Silver Spring)
2. 39th Annual Meeting of the Obesity Society at Obesityweek® November 1-5, 2021: oral and poster abstracts were published as supplement of Obesity (Silver Spring)
3. ENDO 2022 Annual Meeting of the Endocrine Society March 20-23, 2022: oral and poster abstracts were published as supplement of the Journal of the Endocrine Society
4. ENDO 2021 Annual Meeting of the Endocrine Society March 20-23, 2022: oral and poster abstracts were published as supplement of the Journal of the Endocrine Society
5. 82th Scientific Sessions of the American Diabetes Association (ADA) June 3-7, 2022: oral and poster abstracts were published as supplement of Diabetes
6. 81st Scientific Sessions of the American Diabetes Association (ADA) June 25-29, 2021: oral and poster abstracts were published as supplement of Diabetes
7. ZoomForward 2022: European Congress on Obesity (ECO), May 4-7, 2022: oral and poster abstracts were published as supplement of Obesity Facts
8. European Congress on Obesity (ECO Online 2021). 28th Congress, May 10-13, 2021: oral and poster abstracts were published as supplement of Obesity FactsFor grey literature, excerpta Medica Database

**Supplementary Table S2. Search Strategy**

|  | **Search terms** | **Total** | **Embase** | **Ovid** | **Cochrane** |
| --- | --- | --- | --- | --- | --- |
| 1 | Body mass/ or Body mass index/ or waist circumference/ | 812851 | 642664 | 152140 | 18047 |
| 2 | Obesity/ | 761949 | 530416 | 213944 | 17589 |
| 3 | overweight.ti,ab. | 236146 | 131003 | 85883 | 19260 |
| 4 | (obes* or overweigh* or over-weigh* or "body mass index" or BMI or BMIs or bodyweight or decrease in weight or losing weight or loss of weight or (weight* adj1 (loss or lost or maintenance or management or change* or decreas* or reduc* or control))).ti,ab. or Body Weight Loss/ or Weight Change/ or (decrease in weight or losing weight).ti,ab. | 1937992 | 1133729 | 691368 | 112895 |
| 5 | (Malignant neoplasm* or neoplasm* or cancer* or carcinoma*).ti,ab. | 6699585 | 3797134 | 2711846 | 190605 |
| 6 | Bariatric Surgery/ or sleeve gastrectomy/ or gastric banding/ or Gastric Bypass/ | 82140 | 55382 | 25361 | 1397 |
| 7 | (Bariatric surgery or sleeve gastrectomy or gastric banding or Gastric Bypass).ti,ab. | 99363 | 61328 | 33592 | 4443 |
| 8 | lifestyle modification/ or exercise/ or diet/ | 962951 | 612850 | 315310 | 34791 |
| 9 | (life style intervention or lifestyle intervention or life style modification* or lifestyle modification* or exercise* or diet).ti,ab. | 1840011 | 962448 | 718944 | 158619 |
| 10 | Randomized Controlled Trial/ or (randomization or randomisation or randomized controlled trial or Clinical Trial or Double Blind Method or Single Blind Method or multicenter study or clinical trial or Clinical Trials or (clinical adj trial*) or prospective).ti,ab. | 4595908 | 2371222 | 1638425 | 586261 |
| 11 | ((Case control adj (study or studies)) or (Cohort adj (study or studies)) or (follow up adj (study or studies)) or (observational adj (study or studies)) or survey* or (epidemiologic* adj (study or studies))).ti,ab. | 3636076 | 2056170 | 1501805 | 78101 |
| 12 | (prospective or retrospective or longitudinal or literature review or systematic review* or meta anal*).ti,ab. | 5585379 | 3217131 | 2122024 | 246224 |
| 13 | (electronic database* or computeri?ed database* or online database* or registry or registries or claim*).ti,ab. | 882124 | 519730 | 334473 | 27921 |
| 14 | (news or editorial or case reports or letter or comment).pt. or case report.ti. | 6967893 | 2441332 | 4504822 | 21739 |
| 15 | (Case stud* or case report* or case series or case histor* or cross-sectional or cross sectional).ti,ab. | 2803097 | 1594902 | 1183712 | 24483 |
| 16 | review/ or editorial/ or letter/ or News/ or case report/ or case reports/ | 14624086 | 7454118 | 7167628 | 2340 |
| 17 | (child* or newborn or Infant or school child* or adolescent or preschool child* or pregnan* or pediatric).ti,ab. | 6024660 | 3253378 | 2538612 | 232670 |
| 18 | child/ or newborn/ or Infant/ or school child/ or adolescent/ or preschool child/ or pregnancy/ or pediatric/ | 9245241 | 4413421 | 4635941 | 195879 |
| 19 | (after * cancer diagnos* or after* cancer* or cancer survivor* or cancer patient? or patient? with * cancer or people with * cancer).ti. | 257602 | 148083 | 92269 | 17250 |
| 20 | Animal/ or experimental animal/ or animal experiment/ or animal model/ or animal cell culture/ or in vitro study/ | 13152630 | 5880614 | 7269873 | 2143 |
| 21 | 1 or 2 or 3 or 4 | 2252603 | 1387069 | 747466 | 118068 |
| 22 | 6 or 7 or 8 or 9 | 2300929 | 1234240 | 895563 | 171126 |
| 23 | 10 or 11 or 12 or 13 | 10797439 | 5956390 | 4177658 | 663391 |
| 24 | 14 or 15 or 16 or 17 or 18 or 19 or 20 | 35948575 | 17680906 | 17866444 | 401225 |
| 25 | 5 and 21 and 22 | 33822 | 20185 | 11255 | 2382 |
| 26 | 23 and 25 | 14490 | 8313 | 4976 | 1201 |
| 27 | 26 not 24 | 8754 | 5117 | 2829 | 808 |
| 28 | limit 27 to (human and english language and yr="2019 -Current") [Limit not valid in CCTR; records were retained] | 2774 | 1791 | 691 | 292 |
| 29 | remove duplicates from 28 | 2653 | 1673 | 689 | 291 |
| 30 | After removing duplicates from Endnote | 1954 |  |  |  |

**Supplementary Table S3. Preferred Reporting Items for Systematic Reviews and Meta-Analyzes (PRISMA) statement**

| **Section and Topic** | **Item #** | **Checklist item** | **Location where item is reported** |
| --- | --- | --- | --- |
| **TITLE** | | |  |
| Title | 1 | Identify the report as a systematic review. | Yes, Title |
| **ABSTRACT** | | |  |
| Abstract | 2 | See the PRISMA 2020 for Abstracts checklist. | Yes, Abstract |
| **INTRODUCTION** | | |  |
| Rationale | 3 | Describe the rationale for the review in the context of existing knowledge. | Yes |
| Objectives | 4 | Provide an explicit statement of the objective(s) or question(s) the review addresses. | Yes |
| **METHODS** | | |  |
| Eligibility criteria | 5 | Specify the inclusion and exclusion criteria for the review and how studies were grouped for the syntheses. | Yes, Method- Literature screening and eligibility criteria |
| Information sources | 6 | Specify all databases, registers, websites, organisations, reference lists and other sources searched or consulted to identify studies. Specify the date when each source was last searched or consulted. | Yes, Method- first paragraph, Method-publication search section |
| Search strategy | 7 | Present the full search strategies for all databases, registers and websites, including any filters and limits used. | Yes, Method-publication search section, Supplementary Table S 1 |
| Selection process | 8 | Specify the methods used to decide whether a study met the inclusion criteria of the review, including how many reviewers screened each record and each report retrieved, whether they worked independently, and if applicable, details of automation tools used in the process. | Yes, Method- Literature screening and eligibility criteria |
| Data collection process | 9 | Specify the methods used to collect data from reports, including how many reviewers collected data from each report, whether they worked independently, any processes for obtaining or confirming data from study investigators, and if applicable, details of automation tools used in the process. | Yes, Method- study selection, data extraction |
| Data items | 10a | List and define all outcomes for which data were sought. Specify whether all results that were compatible with each outcome domain in each study were sought (e.g. for all measures, time points, analyses), and if not, the methods used to decide which results to collect. | Yes, Method- data extraction |
|  | 10b | List and define all other variables for which data were sought (e.g. participant and intervention characteristics, funding sources). Describe any assumptions made about any missing or unclear information. | Yes, Method- data extraction |
| Study risk of bias assessment | 11 | Specify the methods used to assess risk of bias in the included studies, including details of the tool(s) used, how many reviewers assessed each study and whether they worked independently, and if applicable, details of automation tools used in the process. | Yes, Method- Bias assessment |
| Effect measures | 12 | Specify for each outcome the effect measure(s) (e.g. risk ratio, mean difference) used in the synthesis or presentation of results. | Yes, Literature screening and eligibility criteria |
| Synthesis methods | 13a | Describe the processes used to decide which studies were eligible for each synthesis (e.g. tabulating the study intervention characteristics and comparing against the planned groups for each synthesis (item #5)). | Yes, Literature screening and eligibility criteria |
|  | 13b | Describe any methods required to prepare the data for presentation or synthesis, such as handling of missing summary statistics, or data conversions. | No |
|  | 13c | Describe any methods used to tabulate or visually display results of individual studies and syntheses. | No |
|  | 13d | Describe any methods used to synthesize results and provide a rationale for the choice(s). If meta-analysis was performed, describe the model(s), method(s) to identify the presence and extent of statistical heterogeneity, and software package(s) used. | No |
|  | 13e | Describe any methods used to explore possible causes of heterogeneity among study results (e.g. subgroup analysis, meta-regression). | No |
|  | 13f | Describe any sensitivity analyses conducted to assess robustness of the synthesized results. | No |
| Reporting bias assessment | 14 | Describe any methods used to assess risk of bias due to missing results in a synthesis (arising from reporting biases). | No |
| Certainty assessment | 15 | Describe any methods used to assess certainty (or confidence) in the body of evidence for an outcome. | No |
| **RESULTS** | | |  |
| Study selection | 16a | Describe the results of the search and selection process, from the number of records identified in the search to the number of studies included in the review, ideally using a flow diagram. | Yes, Results -paragraph describing Figure 1 |
|  | 16b | Cite studies that might appear to meet the inclusion criteria, but which were excluded, and explain why they were excluded. | Yes, Results -paragraph describing Figure 1 |
| Study characteristics | 17 | Cite each included study and present its characteristics. | Yes, Table 1 |
| Risk of bias in studies | 18 | Present assessments of risk of bias for each included study. | Yes, Table 2 |
| Results of individual studies | 19 | For all outcomes, present, for each study: (a) summary statistics for each group (where appropriate) and (b) an effect estimate and its precision (e.g. confidence/credible interval), ideally using structured tables or plots. | Yes, Figure 2 |
| Results of syntheses | 20a | For each synthesis, briefly summarise the characteristics and risk of bias among contributing studies. | Yes, Table 2 |
|  | 20b | Present results of all statistical syntheses conducted. If meta-analysis was done, present for each the summary estimate and its precision (e.g. confidence/credible interval) and measures of statistical heterogeneity. If comparing groups, describe the direction of the effect. | Yes, Table 2, Supplementary Table S4A-4L |
|  | 20c | Present results of all investigations of possible causes of heterogeneity among study results. | No |
|  | 20d | Present results of all sensitivity analyses conducted to assess the robustness of the synthesized results. | No |
| Reporting biases | 21 | Present assessments of risk of bias due to missing results (arising from reporting biases) for each synthesis assessed. | No |
| Certainty of evidence | 22 | Present assessments of certainty (or confidence) in the body of evidence for each outcome assessed. | No |
| **DISCUSSION** | | |  |
| Discussion | 23a | Provide a general interpretation of the results in the context of other evidence. | Yes, Discussion |
|  | 23b | Discuss any limitations of the evidence included in the review. | Yes, Discussion-limitation |
|  | 23c | Discuss any limitations of the review processes used. | Yes, Discussion-limitation |
|  | 23d | Discuss implications of the results for practice, policy, and future research. | Yes, Discussion |
| **OTHER INFORMATION** | | |  |
| Registration and protocol | 24a | Provide registration information for the review, including register name and registration number, or state that the review was not registered. | No |
|  | 24b | Indicate where the review protocol can be accessed, or state that a protocol was not prepared. | No |
|  | 24c | Describe and explain any amendments to information provided at registration or in the protocol. | No |
| Support | 25 | Describe sources of financial or non-financial support for the review, and the role of the funders or sponsors in the review. | No |
| Competing interests | 26 | Declare any competing interests of review authors. | Yes, Conflict of Interest |
| Availability of data, code and other materials | 27 | Report which of the following are publicly available and where they can be found: template data collection forms; data extracted from included studies; data used for all analyses; analytic code; any other materials used in the review. | Yes, Data availability statement, Supporting information |

*From:*  Page MJ, McKenzie JE, Bossuyt PM, Boutron I, Hoffmann TC, Mulrow CD, et al. The PRISMA 2020 statement: an updated guideline for reporting systematic reviews. BMJ 2021;372:n71. doi: 10.1136/bmj.n71

For more information, visit: <http://www.prisma-statement.org/>

**Supplementary Table S4A. Association between intentional weight loss and overall cancer incidence, by each study (n=7)**

| **Study** | **Group** | **Cancer case** | **aHR (point estimate [95% CI], p)** | **Incidence rate** | **Other measure** | **Notes** |
| --- | --- | --- | --- | --- | --- | --- |
| **Aminian et, 2022** | BS vs NS | 200 vs.1331 | **0.83 [0.69-0.99]*, P= 0.04)** | 6.3 vs 8.0 per 1,000PY | Cumulative incidence: 0.068[0.057-0.079] vs 0.083[0.078-0.088] Absolute risk difference: 1.5%[0.3%-2.7%] Diff. in rate per 1,000PY: 1.69[0.72-2.66] | BS had significantly lower risk (vs NS) |
| **Wei et al, 2021** | BS vs NS | 11 vs.47 | 1.254[0.649-2.422], p=0.510 | Before trimming: 1.139 [0.568-2.038] vs 0.937 [0.689-1.247] per 100PY After trimming: 1.175 [0.586-2.102] vs. 0.933 [0.683-1.244] per 100PY | Cumulative incidence:  0.032 vs 0.029 | No significant difference |
| **Kao et al, 2021** | BS vs GP | 109 (1.18%) vs 542 (1.46% | 1.00 [0.81-1.23], p=0.9997 | NR | NR | Significance higher risk of new malignancy NS vs GP (BS had no significant difference compared with GP), especially for younger men and female. For age gender subgroups, significant higher risk was found in: **Male, 18-35 y/o (aHR =1.37, p=0.003) Female, 18-35y/o (aHR=1.62 [1.38–1.90], p<0.0001) Female, 35-55 y/o (aHR=1.27 [1.19–1.35], p<0.0001)** |
|  | NS vs GP | 2326/93880 (2.48%) vs 7407/375520(1.97%) | **1.22 (1.16-1.28)*, p<0.0001** | NR | NR |  |
| **Rustgi et al, 2021** | BS vs NS | Total case: 2,823 | **0.82[0.76-0.89]*** | NR | NR | BS had significantly lower risk (vs NS) |
| **Tao et al, 2020^a^** | BS vs NS | 1,314 (2.7%) vs 24,565 (5.2%) | **All years**: **0.89 [0.83–0.94]*** **By length of follow-up:** 0-4 years: 0.79 [0.72-0.87]* 5-9 years: 0.91[0.80-1.03] ≥10 years: 0.98 [0.89-1.07] | NR | **aHR by gender** Women: **0.86[0.80–0.92]*** Men: 0.98[0.95–1.01]) | Overall, BS had significantly lower risk (vs NS); stratified analysis found that decreased risk was statistically significant only in women and not in men and only within the first five years of surgery. |
| **Yeh et al, 2020** | ILI vs DSE | 332 vs 352 | 0.93[0.80-1.08], p=0.32 | 13.2 vs 14.2 per 1,000PY | NR | No significant difference |
| **Schauer et al, 2019** | BS vs NS | 488 vs 2,055 | **0.67[0.60-0.74]*, P < 0.001** | NR | NR | BS had significantly lower risk (vs NS) |

Abbreviations: aHR, adjusted hazard ratio; BS, bariatric surgery intervention; DSE, diabetes support and education; GP, general population; ILI, intensive lifestyle intervention; NR, not reported; NS, nonsurgical (usually the control); PY, person-years.

**Supplementary Table S4B. Association between intentional weight loss and a combination of obesity-associated cancer risk, by each study (n=6)**

| **Study** | **Obesity-associated cancer definition** | **Group** | **Cancer Case** | **aHR (point estimate [95% CI], p)** | **Incidence rate** | **Other measure** | **Notes** |
| --- | --- | --- | --- | --- | --- | --- | --- |
| **Aminian et al, 2022** | Oesophageal adenocarcinoma; renal cell carcinoma; postmenopausal breast cancer (diagnosed at =55 years of age) or breast cancer in younger patients who had bilateral oophorectomy; cancer of the gastric cardia, colon, rectum, liver, gallbladder, pancreas, ovary, corpus uteri, or thyroid; and multiple myeloma. | BS vs NS | 96 vs 780 | **0.68[0.53-0.87]*, p =0.002** | 3.0 vs 4.6 per 1,000PY; | Absolute risk difference: 2.0% [1.2%-2.7%]  Cumulative incidence at year10: 2.9% [2.2%-3.6%] vs 4.9% [4.5%-5.3%] | BS had significantly lower risk (vs NS) |
| **Wei et al, 2021** | Esophagus, gastric cardia, colon, rectum, liver, gallbladder, pancreas, breast [females only], corpus uteri, ovary, kidney, meninges, and multiple myeloma | BS vs NS | 5 vs 32 | 0.843[0.328-2.170], p=0.724 | Before trimming: 0.514 (0.167-1.201) vs 0.636 (0.435-0.898) per 100 PY After trimming: 0.531 (0.172-1.238) vs 0.627 (0.426-0.889) per 100 PY | Cumulative incidence 0.014 vs 0.020 | No significant difference |
| **Rustgi et al, 2021** | colon, Rectal, Postmenopausal breast, Liver, kidney, Oesophageal, pancreatic, ovarian, endometrial, multiple myeloma and meningioma | BS vs NS | Total case 911 | **0.65 [0.56–0.75]*** | NR | NR | BS had significantly lower risk (vs NS) |
| **Tao et al, 2020a** | Esophagus (adenocarcinoma), gastric cardia, colon, rectum, liver, gallbladder, pancreas, breast (postmenopausal), corpus uteri, ovary, kidney (renal cell), meningioma, thyroid and multiple myeloma. | BS vs NS | 706 vs. 12,789 | **All years: 0.89 [0.82–0.97]*** | **NR** | **aHR by gender** Women: **0.86[0.78–0.94]*** Men: 1.04[0.87–1.24]  **By length of follow-up:** 0-4 years: **0.77 [0.67-0.88}*** 5-9 years: 0.91[0.76-1.09] ≥10 years: 1.01 [0.90-1.13] | Overall, BS had significantly lower risk (vs NS); stratified analysis found that decreased risk was statistically significant only in women and not in men and only within the first five years of surgery. |
| **Yeh et al, 2020** | Esophagus, colon, rectum, kidney, pancreas, stomach, liver, gallbladder, thyroid, and multiple myeloma in men and women and additional uterus, ovary, postmenopausal breast in women | ILI vs DSE | 158 vs 185 | 0.84 [0.68-1.04], p=0.10 | DSE 7.3 per 1,000PY; ILI 6.1 per 1,000PY | NR | No significant difference |
| **Schauer et al, 2019** | colon, rectal, endometrial, gallbladder, kidney, liver, multiple myeloma, ovarian, pancreas, postmenopausal breast, and thyroid cancers | BS vs NS | 246 vs 1185 | **0.59[0.51-0.69]*, p< 0.001** | NR | **aHR by gender**  women: **0.58[0.49-0.67]***, p< 0.001 men: 0.70[0.46-1.07] | BS had significantly lower risk (vs NS); significant association was found in women but not in men. |

Abbreviations: aHR, adjusted hazard ratio; BS, bariatric surgery intervention; DSE, diabetes support and education; ILI, intensive lifestyle intervention; NR, not reported; NS, nonsurgical (usually the control); PY, person-years.

**Supplementary Table S4C. Association between intentional weight loss and female breast cancer risk, by each study (n=9)**

| **Study** | **Breast cancer type** | **Group** | **Cancer case** | **aHR (point estimate [95% CI], p)** | **Incidence rate** | **Other measure** | **Notes** |
| --- | --- | --- | --- | --- | --- | --- | --- |
| **Doumouras et al, 2023** | Did not specify pre- or post- menopausal, women only | **NS** [each BMI groups] vs **BS** | [BMI>=35: 135(0.97%) BMI 30-34: 150(1.08%) BMI25-29: 135(0.97%) BMI<25: 128(0.92%)]  vs 103(0.74%) | **at 1-, 2-, 5-year washout:** NS (BMI >=35) vs BS  **1.35 [1.15-1.59]*, p=0.005 1.47[1.23-1.77]*, p=0.008 1.55[1.13-2.12]*, p<0.001** NS (BMI 30-34) vs BS **1.42 [1.21-1.67]*, p<0.001 1.54[1.28-1.85]*, p<0.001 1.63[1.20-2.23]*, p<0.001** NS (BMI 25-29) vs BS **1.25 [1.06-1.49]*, p<0.001 1.32[1.09-1.60]*, p<0.001 1.42[1.03-1.96]*, p<0.001** | NR | **aHR with 1-, 2-, 5-year washout:** NS (all BMI groups) vs BS **1.40[1.18-1.67]*, p<0.001 1.31[1.12-1.53]* p<0.001 1.38[1.21-1.58]*, p<0.001** NS (BMI<25) vs BS 1.07 [0.89-1.28], p=0.10 1.20[0.99-1.47] p=0.48  1.31[0.94-1.82], p=0.19 | NS with overweight or obesity (BMI 25-29, BMI 30-34, BMI>=35) had significantly higher risk (vs BS) with 1-, 2-, 5-year washout. |
| **Aminian et al, 2022** | Post-menopausal | BS vs NS | 21 vs 182 | Reported as not significant [data not available] | 0.65 vs 1.07 per 1,000PY | Diff. in rate per 1,000PY: 0.42[0.10, 0.74] Total breast cancer BS vs NS Case: 39 vs 252 incidence rate: 1.20 vs 1.48 per 1,000PY Diff. in rate per 1,000PY: 0.28[-0.14, 0.70] | No significant difference |
| **Doumouras et al, 2022** | Did not specify pre- or post- menopausal, women only | BS vs NS | 99(0.79%) vs 133 (1.09%) (with 1-year washout) | **at 1-, 2-, 5-year washout: 0.81[0.69–0.95]*, p=0.01** (consistent with 2- or 5-year washout period)  0.77[0.64–0.92]*, p=0.03  0.70[0.52–0.94]*, p=0.01 | NR | NR | BS had significantly lower risk (vs NS) Breast cancer case in BS group had higher proportion diagnosed with low-grade tumors and higher proportion diagnosed with high-grade tumors |
| **Kao et al, 2021** | Did not specify pre- or post- menopausal, women only | BS vs GP | NR | Women aged 18-35:  0.47[0.14–1.55], p= 0.2136 Women aged 35-55: 1.19[0.75–1.89], p= 0.4528 | NR | NR | No significant difference |
|  | Did not specify pre- or post- menopausal, women only | NS vs GP | NR | Women aged 18-35: 0.93[0.66–1.32], p = 0.700 Women aged 35-55: 1.11[0.99–1.24], p= 0.068 | NR | NR | No significant difference |
| **Rustgi et al, 2021** | Post-menopausal (defined as diagnoses at age >=55 years) | BS vs NS | Total case 131 | 1.08 [0.74–1.54] | NR | NR | No significant difference |
| **Tao et al, 2020a** | Did not specify Pre- or post- menopausal, women only | BS vs NS | 179 vs 3,328 | **0.81[0.69–0.95]*** | NR | NR | BS had significantly lower risk (vs NS) |
| **Yeh et al, 2020** | Post-menopausal and women only | ILI vs DSE | 62 vs 78 | 0.78 (0.56, 1.09), p=0.15 | 4.0 vs 5.1 per 1,000PY | NR | No significant difference |
| **Feigelson et al, 2020** | Post-menopausal (defined as women diagnosed at age >=55 years or with bilateral oophorectomy) | BS vs NS | 68 vs 331 | **0.55[0.42–0.72]*, p< 0.001** | NR | NR | BS had significantly lower risk (vs NS) **All female breast cancer BS vs NS:** Case: 133 vs 567 aHR: 0.63[0.52–0.76]*, p< 0.001 **Pre-menopausal BS vs NS:** Case: 65 vs 236 aHR: 0.72[0.54–0.94]*, p=0.02 |
| **Schauer et al, 2019** | Post-menopausal (defined as diagnoses at age >=55 years) | BS vs NS | total 301 | **0.50[0.37, 0.67]*, p< 0.001** | NR | NR | BS had significantly lower risk (vs NS) |

Abbreviations: aHR, adjusted hazard ratio; BMI, body mass index; BS, bariatric surgery intervention; DSE, diabetes support and education; GP, general population; ILI, intensive lifestyle intervention; NR, not reported; NS, nonsurgical (usually the control); PY, person-years.

**Supplementary Table S4D. Association between intentional weight loss and cancer risk for endometrial and ovarian cancer, by each study (n=3 for a combination of female breast and genital organ cancer, n=3 for endometrial cancer, n=3 for ovarian cancer)**

| **Study** | **Cancer type** | **Group** | **Cancer case** | **aHR (point estimate [95% CI], p)** | **Incidence rate** | **Other measure** | **Notes** |
| --- | --- | --- | --- | --- | --- | --- | --- |
| **Wei et al, 2021** | Female breast and genital organ cancers | BS vs NS | 2 vs 13 | 0.757[0.171-3.355], p=0.727 | Before trimming: 0.387[0.047-1.398] vs 0.512[0.273-0.876] per 100PY  After trimming:  0.394[0.048-1.424] vs 0.521[0.277-0.891] per 100PY | Cumulative incidence 0.011 vs 0.017 | No significant difference |
| **Kao et al, 2021** | Reported as "Female genital cancer including uterus, ovary, and cervical cancer" case number not reported | BS vs GP | NR | Women aged 18-35: **3.57[1.72–7.44]*, p = 0.0007** Women aged 35-55: 1.19[0.75–1.89], p= 0.4528 | NR | NR | BS had significant higher risk (vs GP) for younger female but not for older female |
|  | Reported as "Female genital cancer including uterus, ovary, and cervical cancer" case number not reported | NS vs GP | NR | Women aged 18-35: 4.03, [2.83–5.73]*, p < 0.0001  Women aged 35-55: 2.19,[1.87–2.56]*, p< 0.0001 | NR | NR | NS had significant higher risk (vs GP) in both younger and older female |
| **Yeh et al, 2020** | Ovary or Uterine | ILI vs DSE | 16 vs 15 | 1.04[0.52, 2.11], p=0.91 | 1.0 vs 1.0 per 1,000PY | NR | No significant difference |
| **Aminian et al, 2022** | Endometrial | BS vs NS | 16 vs 215 | **0.47[0.27-0.83]*** | 0.49 vs 1.26 per 1,000PY | Diff. in rate per 1,000PY: 0.77[0.48, 1.06] | BS had significantly lower risk (vs NS) |
| **Rustgi et al, 2021** | Endometrial | BS vs NS | Total 135 | **0.49[0.31–0.73]*** | NR | NR | BS had significantly lower risk (vs NS) |
| **Tao et al, 2020a** | Endometrial | BS vs NS | 113 vs 2,050 | **0.69[0.56–0.84]*** | NR | NR | BS had significantly lower risk (vs NS) |
| **Schauer et al, 2019** | Endometrial | BS vs NS | Overall 322 | **0.50[0.37-0.67]*, P < 0.001** | NR | NR | BS had significantly lower risk (vs NS) |
| **Aminian et al, 2022** | Ovarian | BS vs NS | 1 vs 33 | Reported as not significant, data not available | 0.03 vs 0.19 per 1,000PY | Diff. in rate per 1,000PY: 0.16[0.07-0.25] | No significant difference |
| **Rustgi et al, 2021** | Ovarian | BS vs NS | Total 74 | 0.70[0.41–1.15] | NR | NR | No significant difference |
| **Schauer et al, 2019** | Ovarian | BS vs NS | Overall 41 | 0.68[NR}, not significant | NR | NR | No significant difference |

Abbreviations: aHR, adjusted hazard ratio; BS, bariatric surgery intervention; DSE, diabetes support and education; GP, general population; ILI, intensive lifestyle intervention; NR, not reported; NS, nonsurgical (usually the control); PY, person-years.

**Supplementary Table S4E. Association between intentional weight loss and cancer risk for oesophageal cancer and gastric cancer, by each study (n=2 reported a combination of oesophageal, gastric and other digestive organ cancers, n=6 for oesophageal, n=4 for gastric)**

| **Study** | **Cancer type** | **Group** | **Cancer case** | **aHR (point estimate [95% CI], p)** | **Incidence rate** | **Other measure** | **Notes** |
| --- | --- | --- | --- | --- | --- | --- | --- |
| **Wei et al, 2021** | Esophagus, stomach and small intestine caner | BS vs NS | 2 vs 8 | 1.313[0.278-6.192], p=0.744 | Before trimming: 0.202[0.025-0.731] vs 0.158[0.068-0.311] per 100PY After trimming: 0.209[0.025-0.754] vs 0.160[0.069-0.316] per 100PY | Cumulative incidence 0.006 vs 0.005 | No significant difference |
| **Lazzati et al, 2023** | Esophago  gastric | BS vs NS | 83 vs 254 | **0.76[0.59-0.98]*, p= 0.03** | 4.9 vs 6.9 per 100,000PY IRR (NS vs BS): 1.42[1.11-1.82]*, p=0.005 | **Oesophageal cancer BS vs NS:** Case: 26 vs 86 Incidence rate per 100000PY: 1.5 vs 2.3 IRR (NS vs BS): 1.54[0.99-2.38], p=0.05 **Gastric cancer BS vs NS:** Case: 57 vs 168 Incidence rate per 100000PY: 3.3 vs 4.6 **IRR (NS vs BS): 1.37 [1.01-1.85]***, p=0.04 | **For Esophagogastric:** BS had significantly lower risk (vs NS) for oesophagogastric cancer **For oesophageal**: no significant difference **For gastric**: NS had significantly higher risk based on IRR (vs BS) |
| **Aminian et al, 2022** | Oesophageal | BS vs NS | 2 vs 5 | Reported as not significant [data not available] | 0.06 vs 0.03 per 1,000PY | Diff. in rate per 1,000PY: -0.03[-0.12, -0.06] | No significant difference |
| **Rustgi et al, 2021** | Oesophageal | BS vs NS | Total 16 | 0.33[0.06–1.18] | NR | NR | No significant difference |
| **Andalib et al, 2020** | Oesophageal | BS(RYGB) vs. NS | 1 vs 6 | 3.04[0.36–25.8] | 15.4 vs 6.5 per 10,000 PY | IRR: 2.38 [0.05–19.6] | No significant difference |
|  | Oesophageal | BS vs NS | 7 vs 6 | 2.47[0.82–7.45] | 22.3 vs 6.5 per 10,000 PY | IRR: 3.46 [1.00–12.5]* |  |
| **Yeh et al, 2020** | Oesophageal | ILI vs DSE | 4 vs 4 | 0.98[0.25, 3.93], p=0.98 | 0.2 vs 0.2 per 1,000PY | NR | No significant difference |
| **Schauer et al, 2019** | Oesophageal | BS vs NS | 0 vs 16 | NR(0 case in BS) | NR | NR | NR |
| **Aminian et al, 2022** | Gastric | BS vs NS | 0 vs 7 | Reported as not significant [data not available] | 0 vs 0.04 per 1,000PY | For gastric cardia:  Case: 0 vs 3; Incidence: 0 vs 0.02 per 1,000PY | No significant difference |
| **Rustgi et al, 2021** | Gastric | BS vs NS | Total 8 | 0.46 [0.03–2.44] | NR | NR | No significant difference |
| **Yeh et al, 2020** | Gastric | ILI vs DSE | 5 vs 7 | 0.70[0.22, 2.22], p=0.55 | 0.2 vs 0.3 per 1,000PY | NR | No significant difference |

Abbreviations: aHR, adjusted hazard ratio; BS, bariatric surgery intervention; DSE, diabetes support and education; ILI, intensive lifestyle intervention; IRR, incidence rate ratio; NR, not reported; NS, nonsurgical (usually the control); PY, person-years; RYGB, Roux-en-Y gastric bypass.

**Supplementary Table S4F. Association between intentional weight loss and cancer risk for liver cancer and gallbladder cancer, by each study (n=1 for liver or gallbladder, n=5 for liver, n=4 for gallbladder)**

| **Study** | **Cancer type** | **Group** | **Cancer case** | **aHR (point estimate [95% CI], p)** | **Incidence rate** | **Other measure** | **Notes** |
| --- | --- | --- | --- | --- | --- | --- | --- |
| **Yeh et al, 2020** | Gallbladder or Liver | ILI vs DSE | 7 vs 3 | 2.28[0.59, 8.83], p=0.23 | 0.3 vs 0.1 per 1,000PY | NR | No significant difference |
| **Aminian et al, 2022** | Liver | BS vs NS | BS n=3 vs NS n=26 | Reported as not significant [data not available] | BS 0.09 vs NBS 0.15 per 1,000PY; | Diff. in rate per 1,000PY: 0.06[-0.06, 0.18] | No significant difference |
| **Wei et al, 2021** | Liver | BS vs NS | 1 vs 2 | 2.584[0.234-28.512] p=0.447 | Before trimming:  0.102[0.003-0.567] vs 0.039[0.005-0.142] After trimming:  0.105[0.003-0.584] vs 0.040[0.005-0.145] | Cumulative incidence:0.003 vs 0.001 | No significant difference |
| **Kao et al, 2021** | Liver | BS vs GP | NR | Men aged 18-35: 1.14[0.11–11.49], p=0.912 Men aged 35-55: 0.22[0.03–1.65], p=0.140 Women aged 18-35: 4.49[0.89–22.62], p=0.069 Women aged 35-55: 0.41[0.05–3.14], p=0.389 | NR | NR | No significant difference |
|  | Liver | NS vs GP | NR | Men aged 18-35: 0.4[0.16–1.01], p=0.053 Men aged 35-55: 0.94[0.77–1.16], p=0.585 Women aged 18-35: 1.17[0.32–4.3], p=0.814 Women aged 35-55: 1.06[0.78–1.44], p=0.694 | NR | NR | No significant difference |
| **Rustgi et al, 2021** | Liver | BS vs NS | Total 49 | **0.48[0.24–0.89]*** | NR | NR | BS had significantly lower risk (vs NS) |
| **Schauer et al, 2019** | Liver | BS vs NS | Total 20 | 0.30[NR], not significant | NR | NR | No significant difference |
| **Aminian et al, 2022** | Gallbladder | BS vs NS | BS n=0 vs NBS n=5 | NR (0 case in BS) | NS 0.03 per 1,000PY | NR | No significant difference |
| **Wei et al, 2021** | Gallbladder | BS vs NS | 1 vs 1 | 5.010[0.313, 80.295], p=0.258 | Before trimming:  0.101[0.003,0.564] vs 0.020 [0.000,0.110] After trimming:  0.104[0.003-0.581] vs 0.020 [0.001,0.112] | Cumulative incidence: 0.003 vs 0.001 | No significant difference |
| **Rustgi et al, 2021** | Gallbladder | BS vs NS | Total 4 | aHR=0.99 (0.05–12.58) | NR | NR | No significant difference |
| **Schauer et al, 2019** | Gallbladder | BS vs NS | Total 12 | 0.55[NR], not significant | NR | NR | No significant difference |

Abbreviations: aHR, adjusted hazard ratio; BS, bariatric surgery intervention; DSE, diabetes support and education; GP, general population; ILI, intensive lifestyle intervention; NR, not reported; NS, nonsurgical (usually the control); PY, person-years.

**Supplementary Table S4G**. **Association between intentional weight loss and cancer risk for kidney cancer, by each study (n=5)**

| **Study [Author year]** | **Cancer type** | **Group** | **Cancer case** | **aHR (point estimate [95% CI], p)** | **Incidence rate** | **Other measure** | **Notes** |
| --- | --- | --- | --- | --- | --- | --- | --- |
| **Aminian et al, 2022** | Kidney | BS vs NS | 10 vs 75 | Reported as not significant [data not available] | 0.31 vs 0.44 per 1,000PY | Diff. in rate per 1,000PY: 0.13[-0.09, 0.35] | No significant difference |
| **Rustgi et al, 2021** | Kidney | BS vs NS | Total 120 | 0.90[0.60–1.32] | NR | NR | No significant difference |
| **Tao et al, 2020a** | Kidney | BS vs NS | 87 vs 990 | **1.44[1.13–1.84]*** | NR | **aHR by gender** Men: **1.53[1.05,2.23]*** Women: **1.39[1.01,1.90]*** | BS vs NBS Significant increased risk for both sexes. |
| **Yeh et al, 2020** | Kidney | ILI vs DSE | 11 vs 17 | 0.63[0.3, 1.35], p=0.24 | 0.4 vs 0.7 per 1,000PY | NR | No significant difference |
| **Schauer et al, 2019** | Kidney | BS vs NS | Total 119 | 0.79[NR], not significant | NR | NR | No significant difference |

Abbreviations: aHR, adjusted hazard ratio; BS, bariatric surgery intervention; DSE, diabetes support and education; ILI, intensive lifestyle intervention; NR, not reported; NS, nonsurgical (usually the control); PY, person-years

**Supplementary Table S4H**. **Association between intentional weight loss and cancer risk for pancreatic cancer, by each study (n=7)**

| **Study [Author year]** | **Cancer type** | **Group** | **Cancer case** | **aHR (point estimate [95% CI], p)** | **Incidence rate** | **Other measure** | **Notes** |
| --- | --- | --- | --- | --- | --- | --- | --- |
| **Bulsei et al, 2022** | Pancreatic | BS vs NS | Total 118 (0.07%) vs 4478(0.35%) | **0.567[0.467,0.689]*, p < 0.0001** | 0.14[0.12,0.17] vs 0.64[0.62,0.66] per 1,000PY | NR | BS had significantly lower risk (vs NS) |
| **Aminian et al, 2022** | Pancreatic | BS vs NS | 4 vs 30 | Reported as not significant [data not available] | 0.12 vs 0.18 per 1,000PY | Diff. in rate per 1,000PY: | No significant difference |
| **Wei et al, 2021** | Pancreatic | BS vs NS | 0 vs 2 | NR (0 case in BS) | Before trimming: 0.039[0.005,0.142] per 100PY After trimming: 0.020[0.001,0.112] per 100PY | Cumulative incidence: 0.001 | NR |
| **Rustgi et al, 2021** | Pancreatic | BS vs NS | Total 44 | **0.46[0.21,0.93]*** | NR | NR | BS had significantly lower risk (vs NS) |
| **Tao et al, 2020a** | Pancreatic | BS vs NS | 41 vs 808 | 1.10[0.78,1.57] | NR | **aHR by gender** Men: 1.00[0.53,1.87] Women: 1.16[0.76,1.77] | No significant difference |
| **Yeh et al, 2020 /LOOK AHEAD** | Pancreatic | ILI vs DSE | 11 vs 20 | 0.55[0.26,1.14], p=0.11 | 0.4 vs 0.8 per 1,000PY | NR | No significant difference |
| **Schauer et al, 2019** | Pancreatic | BS vs NS | Total 47 | **0.46,[0.22,0.97]*, p=0.04** | NR | NR | BS had significantly lower risk (vs NS) |

Abbreviations: aHR, adjusted hazard ratio; BS, bariatric surgery intervention; DSE, diabetes support and education; GP, general population; ILI, intensive lifestyle intervention; NR, not reported; NS, nonsurgical (usually the control); PY, person-years.

**Supplementary Table S4I**. **Association between intentional weight loss and cancer risk for colorectal cancer, by each study (CRC: n=7, colon: n=5[4 with relative incidence measure], rectum/anus: n=4[3 with relative incidence measure])**

| **Study** | **Cancer type** | **Group** | **Cancer case** | **aHR (point estimate [95% CI], p)** | **Incidence rate** | **Other measure** | **Notes** |
| --- | --- | --- | --- | --- | --- | --- | --- |
| **Hussan et al, 2022** | CRC | BS vs NS | 88 vs 325 | Women: 0.81[0.57,1.17], p=0.27 Men: 1.57[0.94,2.66], p=0.09 | 35.9 vs 43.1 per 100,000 PY | **By gender and BS procedure:** Women: **RYGB: 0.40[0.18,0.87]*, p=0.02** SG: 1.04[0.68,1.58], p=0.87 Men: RYGB: 2.04[0.80,5.18], p=0.13 SG: 1.36[0.73,2.57], p=0.34 | Only significant lower risk found in female with RYGB BS vs control. no significant difference for all bariatric surgery in female or male; results remain the same even after restricted to those with 3+ years follow-up |
| **Aminian et al, 2022** | CRC | BS vs NS | BS n=16 vs NBS n=86 | Reported as not significant [data not available] | 0.49 vs0.50 per 1,000PY | Diff. in rate per 1,000PY: 0.01[-0.25,0.27] | No significant difference |
| **Ciccioriccio et al, 2021** | CRC | BS vs NS | 22(0.10%) | NR | NR | SIR < 1 for SG across both gender and for RYGB in female: Male, RYGB: 1.07[0.91,1.2] Male, SG: **0.5[0.2,0.72]*** Female, RYGB: **0.8[0.32,0.94]*** Female,SG: **0.6[0.3,0.76]*** | BS had significantly lower risk (vs NS), except for male who had RYGB procedure |
| **Rustgi et al, 2021** | CRC, colon & rectum | BS vs NS | Total 131 (colon 116, rectum 15) | CRC: **0.62[0.4,0.93]*** Colon: 0.66 [0.42,1.00] Rectum: 0.44 (0.10,1.37) | NR | NR | BS had significantly lower risk (vs NS) |
| **Taube et al, 2021** | CRC, colon & rectum | BS vs NS | CRC:58 vs 67 Colon:43 vs 40  Rectum:19 vs 31 | CRC:0.89[0.62,1.29],p=0.551 Colon: 1.14[0.72,1.80], p=0.572 Rectum:0.61[0.34,1.10], p=0.099 | NR | NR | No significant difference for colorectal, colon or rectal cancer |
| **Yeh et al, 2020** | CRC | ILI vs DSE | 28 vs 30 | 0.92[0.55, 1.53], p=0.74 | 1.1 vs 1.2 per 1,000PY | NR | No significant difference |
| **Bailly et al, 2020** | CRC | BS vs NS | BS:423 vs 12629 | **0.68 [0.60, 0.77]*** | NR | BS vs GP: 1.0 (95%CI, 0.90-1.09); SIR NS control vs GP:1.34 (95%CI, 1.32-1.36) | BS had significantly lower risk (vs NS). Additionally, SIRs are found higher in men vs women, younger vs older **for both BS and NS**. |
| **Wei et al, 2020** | Colon & rectum | BS vs NS | Colon: 0 vs 6 Rectum/anus 0 vs 3 | NR (0 case in BS) | **NS. Colon** Before trimming:  0.118[0.043, 0.258] per 100PY After trimming:  0.120[0.044, 0.262] per 100PY **NS, Rectal/anus** Before trimming:  0.158[0.068,0.311] per 100PY After trimming:  0.160[0.069,0.316] per 100PY | **Cumulative incidence for NS:**  Colon: 0.004 Rectum/anus: 0.002 | NR |
| **Kao et al, 2021** | Colon | BS vs GP | NR | Men aged 18-35:  NR (0 case in BS) Men aged 35-55: 1.28[0.43,3.86], p=0.656 Women aged 18-35: 0.87[0.1,7.22], p=0.896 Women aged 35-55: 1.04[0.39,2.75], p=0.935 | NR | NR | No significant difference across age & gender subgroups |
|  |  | NS vs GP | NR | Men aged 18-35: 0.95[0.46,1.98], p=0.892 Men aged 35-55: 1.15[0.92,1.43], p=0.231 Women aged 18-35: 1.39[0.72,2.7], p=0.330 Women aged 35-55: 1.02[0.83,1.25], p=0.861 | NR | NR |  |
| **Tao et al, 2020^a^** | Colon | BS vs NS | 99 vs 2,066 | 1.12[0.90,1.39] | NR | **aHR by gender** Men:1.11[0.78-1.59] Women:1.13[0.86,1.48] | No significant difference |
|  | Rectum &anus | BS vs NS | 45 vs 913 | 1.07[0.77,1.48] | NR | Men:1.09[0.67,1.76] Women:1.05[0.68,1.63] | No significant difference |

Abbreviations: aHR, adjusted hazard ratio; BS, bariatric surgery intervention; CRC, colorectal cancer; DSE, diabetes support and education; GP, general population; ILI, intensive lifestyle intervention; NR, not reported; NS, nonsurgical (usually the control); PY, person-years; RYGB, Roux-en-Y gastric bypass; SIR, standardized incidence ratio; SG, sleeve gastrectomy.

**Supplementary Table S4J**. **Association between intentional weight loss and cancer risk for thyroid cancer, by each study (n=5)**

| **Study** | **Cancer type** | **Group** | **Cancer case** | **aHR (point estimate [95% CI], p)** | **Incidence rate** | **Other measure** | **Notes** |
| --- | --- | --- | --- | --- | --- | --- | --- |
| **Aminian et al, 2022** | Thyroid | BS vs NS | BS n=21 vs NS n=110 | Reported as not significant [data not available] | 0.65 vs 0.64 per 1,000PY | Diff. in rate per 1,000PY: -0.01[-0.31,0.29] | No significant difference |
| **Rustgi et al, 2021** | Thyroid | BS vs NS | Total 143 | **0.61[0.41,0.89]*** | NR | NR | BS had significantly lower risk (vs NS) |
| **Tao et al, 2020a** | Thyroid | BS vs NS | 20 vs 300 | 0.72[0.44,1.17] | NR | **aHR by gender** Men: 0.21[0.03,1.55] Women: 0.84[0.50,1.39] | No significant difference |
| **Yeh et al, 2020** | Thyroid | ILI vs DSE | 7 vs 10 | 0.69[0.26, 1.8], p=0.44 | 0.4 vs 0.3 per 1,000PY | NR | No significant difference |
| **Schauer et al, 2019** | Thyroid | BS vs NS | Total 95 | 0.92[NR], not significant | NR | NR | No significant difference |

Abbreviations: aHR, adjusted hazard ratio; BS, bariatric surgery intervention; DSE, diabetes support and education; ILI, intensive lifestyle intervention; NR, not reported; NS, nonsurgical (usually the control); PY, person-years.

**Supplementary Table S4K**. **Association between intentional weight loss and cancer risk for multiple myeloma, by each study (n=4)**

| **Study** | **Cancer type** | **Group** | **Cancer case** | **aHR (point estimate [95% CI], p)** | **Incidence rate** | **Other measure** | **Notes** |
| --- | --- | --- | --- | --- | --- | --- | --- |
| **Aminian et al, 2022** | Multiple myeloma | BS vs NS | 5 vs 22 | Reported as not significant [data not available] | 0.15 vs 0.13 per 1,000PY | Diff. in rate per 1,000PY:-0.02[-0.16, 0.12] | No significant difference |
| **Rustgi et al, 2021** | Multiple myeloma | BS vs NS | Total 50 | **aHR=0.33 (0.14–0.69)*** | NR | NR | BS had significantly lower risk (vs NS) |
| **Yeh et al, 2020** | Multiple myeloma | ILI vs DSE | 7 vs 1 | 6.88 (0.85-55.96), p=0.07 | 0.3 vs 0.0 per 1,000PY | NR | No significant difference |
| **Schauer et al, 2019** | Multiple myeloma | BS vs NS | Total 18 | 0.50[NR], not significant | NR | NR | No significant difference |

Abbreviations: aHR, adjusted hazard ratio; BS, bariatric surgery intervention; DSE, diabetes support and education; ILI, intensive lifestyle intervention; NR, not reported; NS, nonsurgical (usually the control); PY, person-years.

**Supplementary Table S4L**. **Association between intentional weight loss and cancer risk for meningioma, by each study (n=2)**

| **Study** | **Cancer type** | **Group** | **Cancer case** | **aHR (point estimate [95% CI], p)** | **Incidence rate** | **Other measure** | **Notes** |
| --- | --- | --- | --- | --- | --- | --- | --- |
| **Aminian et al, 2022** | Meningioma | BS vs NS | 7 vs 57 | Reported as not significant [data not available] | 0.22 vs 0.33 per 1,000PY | Diff. in rate per 1,000PY: 0.11[-0.07, 0.29] | No significant difference |
| **Rustgi et al, 2021** | Meningioma | BS vs NS | Total 6 | 0.52[0.05–2.90] | NR | NR | No significant difference |

Abbreviations, aHR: adjusted hazard ratio; BS: bariatric surgery intervention; NR: not reported; NS: nonsurgical (usually the control).
